# An EMG Interface for the Control of Motion and Compliance of a Supernumerary Robotic Finger

**DOI:** 10.3389/fnbot.2016.00018

**Published:** 2016-11-11

**Authors:** Irfan Hussain, Giovanni Spagnoletti, Gionata Salvietti, Domenico Prattichizzo

**Affiliations:** ^1^Department of Information Engineering and Mathematics, Università degli Studi Siena, Siena, Italy; ^2^Department of Advanced Robotics, Istituto Italiano di Tecnologia, Genoa, Italy

**Keywords:** wearable robotics, supernumerary robotic fingers, compliance control

## Abstract

In this paper, we propose a novel electromyographic (EMG) control interface to control motion and joints compliance of a supernumerary robotic finger. The supernumerary robotic fingers are a recently introduced class of wearable robotics that provides users additional robotic limbs in order to compensate or augment the existing abilities of natural limbs without substituting them. Since supernumerary robotic fingers are supposed to closely interact and perform actions in synergy with the human limbs, the control principles of extra finger should have similar behavior as human’s ones including the ability of regulating the compliance. So that, it is important to propose a control interface and to consider the actuators and sensing capabilities of the robotic extra finger compatible to implement stiffness regulation control techniques. We propose EMG interface and a control approach to regulate the compliance of the device through servo actuators. In particular, we use a commercial EMG armband for gesture recognition to be associated with the motion control of the robotic device and surface one channel EMG electrodes interface to regulate the compliance of the robotic device. We also present an updated version of a robotic extra finger where the adduction/abduction motion is realized through ball bearing and spur gears mechanism. We have validated the proposed interface with two sets of experiments related to compensation and augmentation. In the first set of experiments, different bimanual tasks have been performed with the help of the robotic device and simulating a paretic hand since this novel wearable system can be used to compensate the missing grasping abilities in chronic stroke patients. In the second set, the robotic extra finger is used to enlarge the workspace and manipulation capability of healthy hands. In both sets, the same EMG control interface has been used. The obtained results demonstrate that the proposed control interface is intuitive and can successfully be used, not only to control the motion of a supernumerary robotic finger but also to regulate its compliance. The proposed approach can be exploited also for the control of different wearable devices that has to actively cooperate with the human limbs.

## 1 Introduction

Wearable robotic devices have been mainly used in substitution of lost limbs [e.g., prosthetic limbs (Carrozza et al., 2004)] or for human limb rehabilitation [e.g., exoskeletons (Pons, 2008)]. Besides traditional wearable robotic structures, a very promising research direction aims at adding robotic extra limbs to humans, rather than substituting or enhancing the human limbs (Davenport et al., 2012; Wu and Asada, 2014). The advantage of using wearable robotic extra limbs is twofold. From one side, this addition can enable humans to augment their capabilities (Llorens-Bonilla et al., 2012). On the other side, extra limbs can compensate the missing abilities of impaired limbs, e.g., in case of chronic stroke patients (Salvietti et al., 2016).

We recently started to investigate how an extra (supernumerary) robotic finger can be used in cooperation with the human hand. We mostly focus on two possible applications: compensate the missing abilities of stroke patients with a paretic hand and augment the human healthy hand so as to enhance its capabilities. Concerning grasp compensation in stroke patient, we noted that, in last decade, many wearable devices have been proposed, especially for hand rehabilitation and functional recovery (Heo et al., 2012; Lum et al., 2012). However, only 5–20% of patients show a complete recover of upper limb 6 months after the stroke (Nakayama et al., 1994). We have, thus, proposed a wearable extra finger device that allows the patient to regain the grasping function of the hand when the deficit is stabilized (Hussain et al., 2015b; Salvietti et al., 2016). The main idea was to have the robotic finger and paretic arm acting as the two parts of a gripper to hold an object. The human user was able to control the flexion/extension of the robotic finger through a switch placed on a ring, while being provided with vibrotactile feedback about the forces exerted by the robotic finger on the grasped object. In Salvietti et al. (2016), we introduced an EMG interface that captures the frontalis muscle activation to control the finger flexion/extension. Finally, in Hussain et al. (2016), we proposed an underactuated compliant extra finger as well as an EMG interface embedded in a cap. Concerning augmenting human healthy hand, in Prattichizzo et al. (2014a), we presented a preliminary version of a robotic extra finger showing how this wearable device is able to enhance grasping capabilities and hand dexterity in healthy subjects. In Prattichizzo et al. (2014b), we presented an object-based mapping algorithm to control robotic extra limbs without requiring explicit commands by the user. The main idea of the mapping was to track human hand by means of dataglove and reproduce the main motions on the extra finger. Although the earlier presented works on extra-robotic fingers clearly report the impact of the research, the presented robotic devices and their control interfaces are not enough general. In fact, the proposed systems could manage only few inputs (e.g., few predefined closing trajectories), and no solutions have been proposed to modulate the compliance of the robotic finger so as to control the force on the grasped object. Since supernumerary robotic fingers are supposed to closely interact and perform actions in synergy with the human limbs, the control principles of extra finger should have similar behavior as human’s ones. Humans can dynamically change their arm stiffness depending on the environment and the tasks being executed (Ajoudani et al., 2012). For instance, stiffness can be increased by muscle cocontraction when we want to make a precise positioning, or when we hold heavy loads. So that, making the actuators and sensing capabilities of the robotic extra finger compatible to implement stiffness regulation control techniques is of primary importance (Hogan, 1985). Second, we believe that the user should directly control through an interfaces of the stiffness of robotic fingers.

The main contribution of this work is the development of a novel EMG interface that can be used to control both the motion of the supernumerary robotic finger and its compliance and thus the tightness of the obtained grasp. In particular, we relate different finger motions to different gestures of the human hand. We used a commercial EMG interface (Myo Armband, ThalmicLab) for hand gesture recognition. For the compliance control, we used a dedicated surface one bipolar EMG channel to read the user biceps signal. The separation of the two EMG reading allows the user to better control independently grasp tightness and device motion. We also present an updated version of the prototype of robotic extra finger where the adduction/abduction motion is realized through ball bearing and spur gears mechanism. The proposed system can be used both by patients for grasp compensation and by healthy subjects for grasp augmentation. We performed a pilot study to demonstrate the feasibility of the approach both with healthy hand for augmenting its abilities and simulated paretic hand to compensate missing grasp abilities. We involved four healthy subjects to perform two different sets of experiments involving the augmentation of a healthy hand or the compensation of a simulated paretic hand. In both cases, the interface resulted sufficient to effectively control the extra-robotic finger so as to fulfill the proposed task. In all the experiments, the wearable device was worn in one arm, whereas the control interface was worn on the other. In fact, while healthy subjects could potentially wear the interface on the same arm where the device is worn, patients cannot properly control hand motion and muscle contraction in their paretic upper limbs. Use the healthy arm is a possible solution as well as delocalizes the EMG reading in another part of the body, see, e.g., Hussain et al. (2016). Note that the hand gestures are necessary only to select a predefined behavior of the device, so it is not necessary to keep a certain gesture for long period. This is important in bimanual tasks where both hands can be used.

The rest of the paper is organized as follows. In Section 2, we present the materials and methods. In particular, the details of the design and development of the proposed supernumerary robotic finger and the proposed EMG control interfaces are explained in details. In Section 3, the experiments using the proposed system are presented. The results are detailed in Section 4 and discussed in Section 5. Finally, in Section 6, conclusion and future work are outlined.

## 2 Materials and Methods

### 2.1 The Supernumerary Robotic Finger

The proposed supernumerary robotic finger is composed of modules connected to partially resemble the human finger mechanical structure. Human hand fingers, excluding the thumb, consist of four phalanges connected by three joints (Jones and Lederman, 2006). The structure of the thumb is different since it has two joints at the base for the anterposition or retroposition combined with the radial or palmar abduction motions. The other fingers are capable of both adduction–abduction and flexion–extension motions. The finger’s kinematic model is typically approximated by using simple revolute joints. This approximation is an effective means of modeling, as these are, in fact, the same as compared to proximal and distal joints of humans. The proximal and distal interphalangeal articulations can have only flexion/extension motion capabilities and typically are represented with a single DoF revolute joint. The metacarpal joints have both adduction/abduction and flexion/extension motion capabilities and can be modeled as a 2-DoFs joint that is composed of two revolute joints with orthogonal rotation axis (universal joint). We designed the kinematic structure of the robotic extra finger such that one motor is adopted to actuate each DoF of the robotic finger so as to replicate the flexion/extension motion of the human finger. While, at the robotic finger base, two motors realize the adduction–abduction and flexion–extension motion to replicate metacarpal joint. We used four modules in a pitch–pitch configuration for the flexion–extension motion of the finger so as to approximate the average length of the whole hand (Taylor and Schwarz, 1955). The adduction/abduction motion of base joint is obtained using spur gears that allows to transmit motion and power. One of the spur gear is mounted on the shaft of the servo motor, whereas the other is placed on the base of the finger. We used bearings to decrease the friction during rotation.

**Figure 1:**
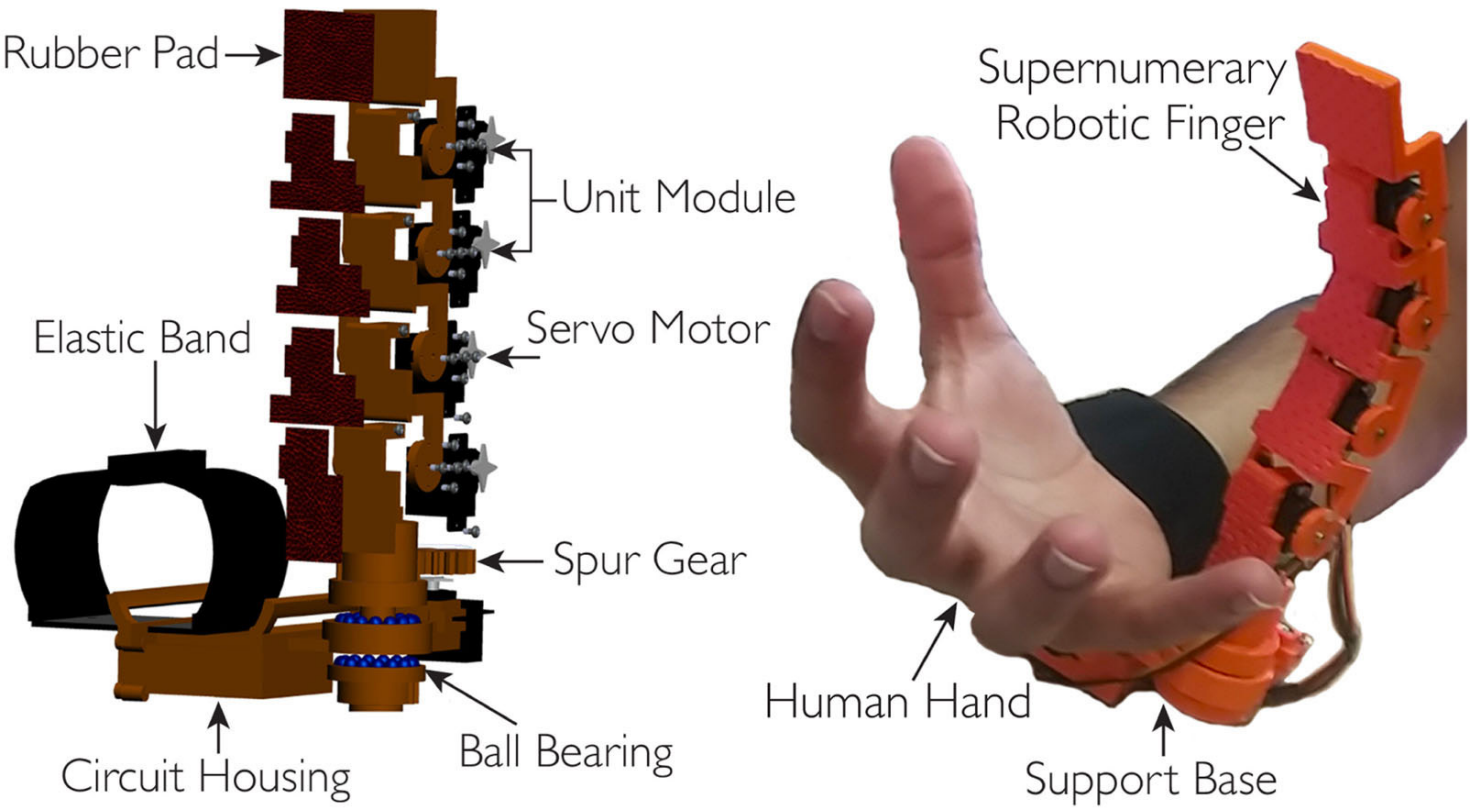
**On left, the exploded cad view, whereas on right, the prototype of the robotic extra finger**. Four modules are used for the flexion/extension motion, while the revolute joint based on bearings and spur gears mechanism at the finger base is used for the adduction/abduction motion. The device can be worn on the forearm through an elastic band.

The finger design is based on the principle of modularity. Each module consists of a servomotor, a 3D printed structure (Acrylonitrile Butadiene Styrene, ABSPlus, Stratasys, USA) and a soft rubber part mounted on front to increase the friction at the contact area. The actuators used are the HS55 MicroLite servo motors. The modules are connected so that one extremity of each module is rigidly coupled with the shaft of the motor through screws, while the other has a pin joint acting as revolute joint. The exploded view and the prototype of the device are shown in Figure 1.

The servo motors are pulse width modulation (PWM) controlled. The PWM signals are generated by a microcontroller At-mega 328 installed on an arduino nano board. The portability and wearability of the device is improved by enclosing all the electronics circuitry in a 3D printed housing which is attached to the finger base support. An external battery pack (5 V) is used to provide power to the actuators. Technical details on the device are summarized in Table 1.

**Table 1:** **The technical details of supernumerary robotic finger**.

Device weight	0.16 kg
Module dimension	42 mm × 33 mm × 20 mm
Module weight	16 g
Support base dimension	78 mm × 24 mm × 5 mm
Support base weight	28 g
Max torque per motor	0.15 Nm
Max payload	0.61 kg
Velocity of one module	0.5 rad/s
External battery pack	5 V

### 2.2 The EMG Control Interface for the Supernumerary Robotic Finger

**Figure 2:**
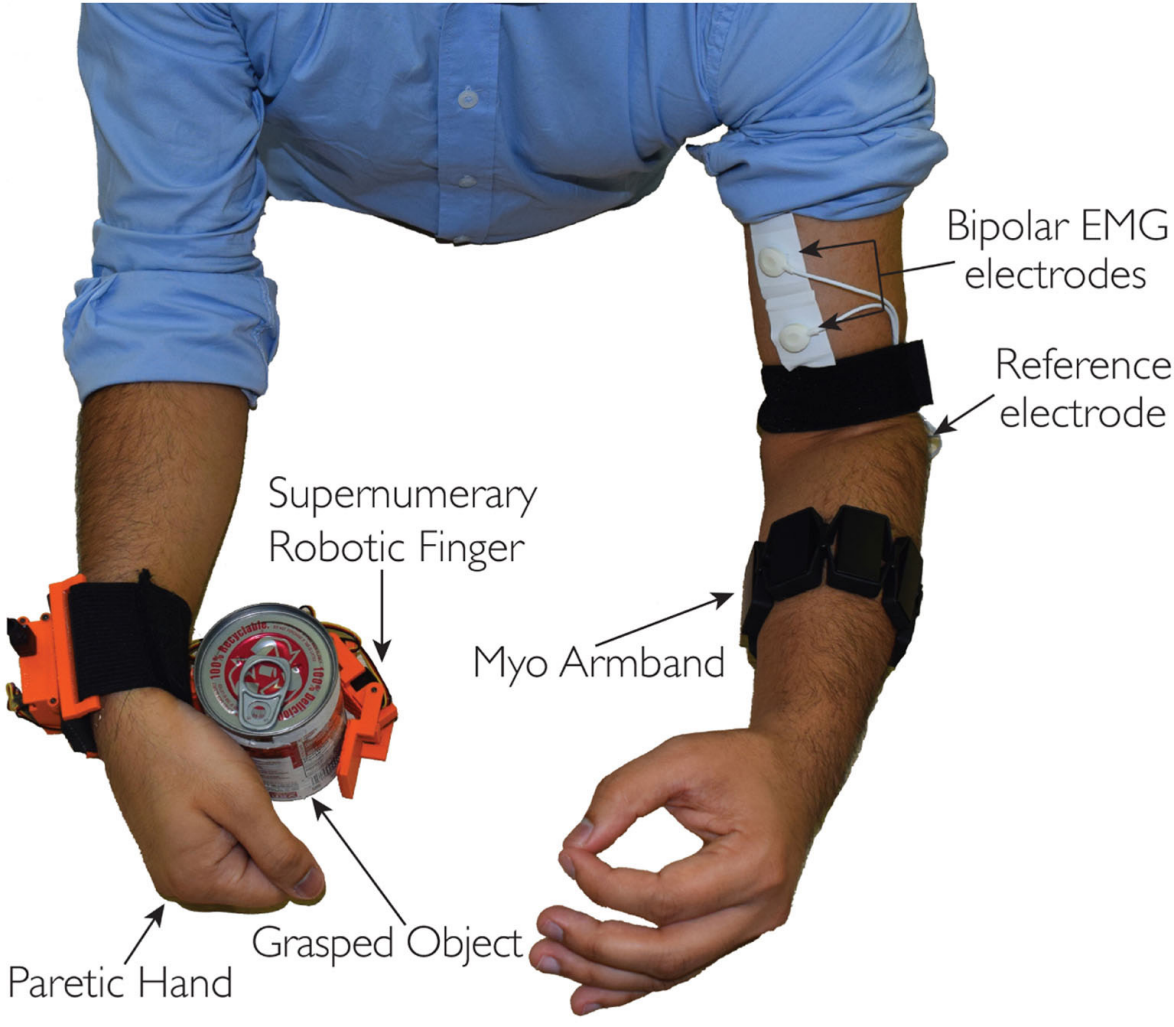
**The complete system: the EMG interface on one arm, whereas the supernumerary robotic finger is on the other arm**. Myo Armband is positioned on the forearm, while the one channel interface is placed on the biceps muscle.

As explained in the introduction, we combined the EMG signals associated with the activation of more muscles for the proposed interface. In particular, we used two EMG interfaces on the arm, one to record the continuous EMG amplitude aiming to regulate the compliance of the device and the second to recognize different hand gestures to be associated with the motion of the robotic finger. Both EMG interfaces are placed on one of the arms, one at the biceps and other at the forearm, while having the robotic finger on the other arm as shown in Figure 2. We developed the circuit acquisition and signal conditioning board for one channel EMG electrodes to measure continuously the biceps muscle EMG signal variations. We used the Myo Armband to recognize the gestures at forearm position.

**Figure 3:**
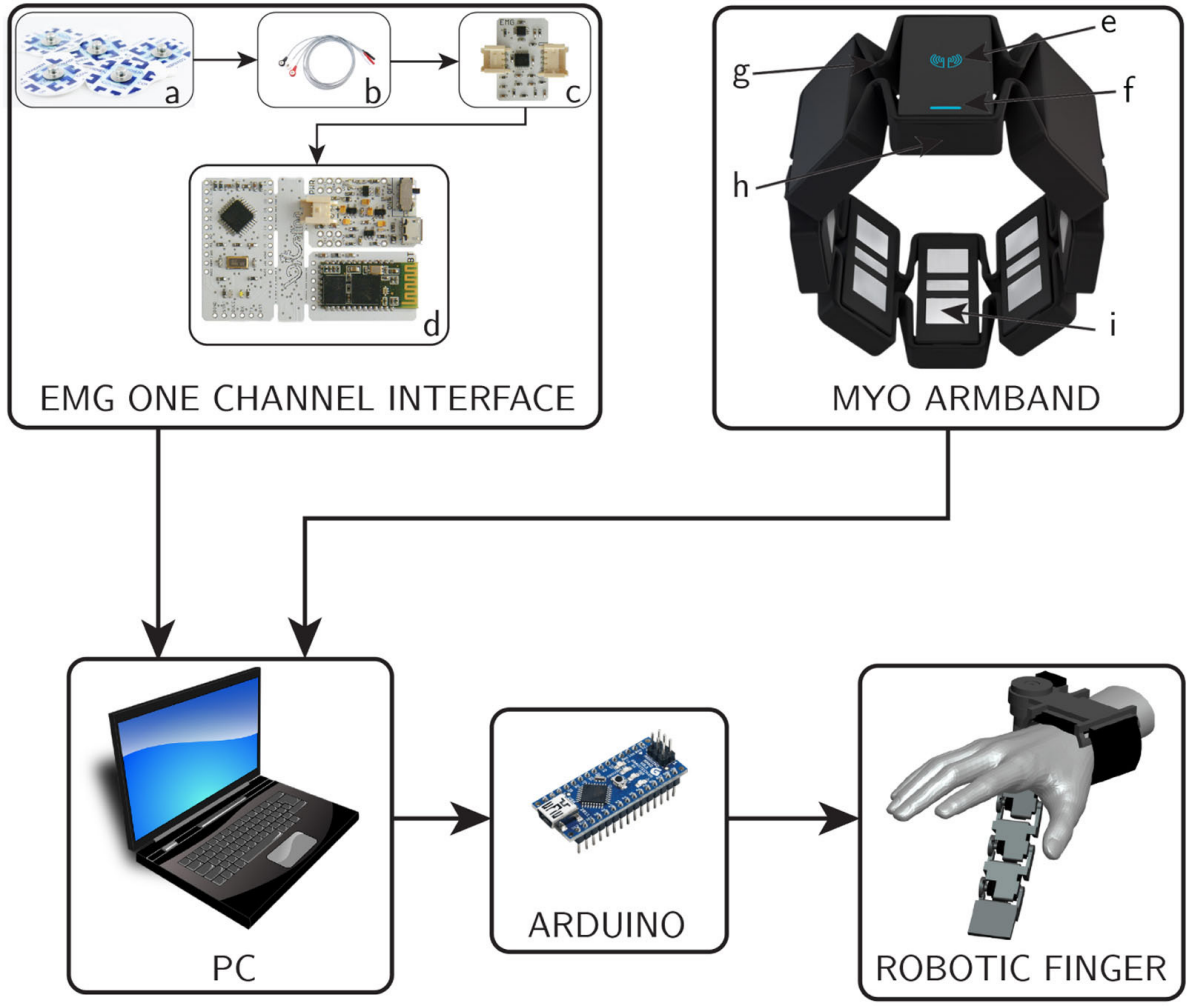
**Block diagram of complete system**. On top left, the block diagram of EMG one channel interface is shown, where (a) surface electrodes, (b) snap leads, (c) acquisition board, and (d) control board. On top, right, the myoarm band with its major components (e) logo LED, (f) status LED, (g) expandable flex, (h) micro USB charging port, and (i) electrical sensor.

Figure 3 shows the block diagram of the proposed system. Both, EMG one channel interface and Myo Armband are connected to a computer through Bluetooth communication. The PC runs MATLAB that is used to process the EMG signal for the compliance control. In order to stream data from Myo Armband to the robotic finger, we used MyoMex. The PC communicates with the robotic device controller (Arduino) through serial communication which in turn controls the motion and compliance of the supernumerary robotic finger.

Section 2.2.1 describes the development of the acquisition and signal conditioning board for one channel EMG interface followed by the compliance regulation of the robotic device through the amplitude variation in the acquired biceps EMG signal. In Section 2.2.2, we describe the gesture recognition through the Myo Armband and their association with the motion control of the supernumerary robotic finger through a finite state machine (FSM).

#### 2.2.1 One Channel EMG Electrodes Interface and Robotic Device Compliance Regulation

**Figure 4:**
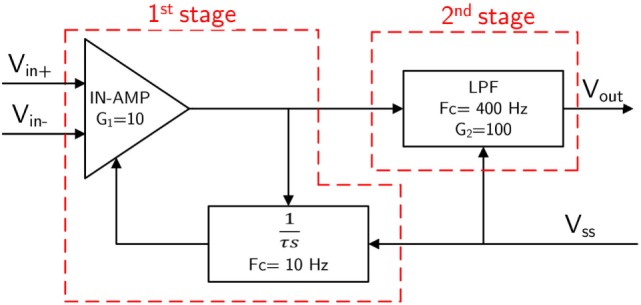
**Block diagram of the EMG circuit board (*Gain* = 1000; *Bandwidth* = 10–400 Hz)**. *V_IN+_* and *V_IN_*_−_ are the “detecting electrodes” while *V_ss_* = *V_cc_*/2 is the “ground electrode.”

We used non-gelled reusable silver/silver-chloride electrodes for the EMG one channel interface. These are recommended for biopotentials recording since they present the lowest noise interface (Merletti et al., 2009). The design and development of the EMG signal acquisition board is carried out, while considering the requirements associated with bandwidth, dynamic range, and physiological principles. The typical EMG waveform is characterized with a spectral content between 10 and 250 Hz with amplitude up to 5 mV, depending on the particular muscle (Merlo and Campanini, 2010). The first stage of the signal conditioning board is developed by using an instrumentation amplifier (INA333) which offers an high common-mode rejection ratio (110 dB @ *G* ≥ 10), while the second stage contains a low-noise high speed operational amplifier (AD869x) to perform band-pass filtering and amplification of the acquired EMG signal. Figure 4 shows the block diagram of the implemented EMG circuit board. Three electrodes are interfaced to the board; two of them (*V_IN+_* and *V_IN_*_−_) are connected to the inputs of an instrumentation amplifier (In-Amp) and third one called “reference electrode” is connected to a mid-supply reference voltage (*V_ss_* = 1.65 V). This configuration improves the quality of EMG signal acquisition as it increases the common-mode rejection ratio (CMRR). The first stage of the EMG board is an In-Amp with an additional stage of AC coupling. This configuration allows a precise control of DC levels rejecting undesired DC offset voltage introduced by electrode–skin interface. The DC component is subtracted by feeding the output signal back to the reference input of the In-Amp, by an integrator feedback network, which results in the first-order high-pass response. The second stage of the EMG board is a 4th order low-pass Butterworth filter. An active topology (a Sallen–Key circuit implementation – 4th order low-pass filter cascading two stages of 2nd order) was chosen to get a better performance and less complexity than a passive one. The acquired EMG signal is sampled at 1 kHz (double EMG band) to avoid aliasing.

**Figure 5:**
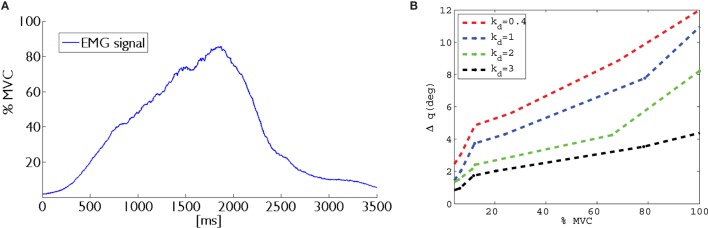
**On left, (A) the maximum voluntary contraction (MVC) proportional to the biceps muscle contraction is shown**. While on right, **(B)** the graph between Δ*q* and percentage of MVC for different values of *k_d_* is plotted.

The reference value of received EMG was normalized using maximum voluntary contraction (MVC) technique (Farina and Merletti, 2000). This solution avoids the problems related to the high influence of detection condition on EMG signal amplitude. In fact, amplitude can greatly vary between electrode sites, subjects, and even day-to-day measures of the same muscle site. We implemented an autotuning procedure based on the MVC in order to better match the user-dependent nature of the EMG signal. The implemented MVC routine consists of a 3-s time window in which the user slowly starts increasing the contraction of the biceps muscle to reach their maximum effort. Figure 5A shows the relation between EMG (percentage of MVC) signal at biceps and time (milliseconds). The relationship between EMG signal and muscle tension is non-linear. The MVC value itself is not calculated as a single peak data point because that would allow too much variability. In order to obtain a more stable reference value, we have implemented an algorithm using a sliding window technique of 500 ms duration to compute the mean amplitude of the highest signal portion acquired during the 3-s time window.

The technical details of the EMG acquisition board are listed in Table 2.

**Table 2:** **Technical details of EMG signal acquisition and conditioning board**.

EMG acquisition box dimensions	3.5 cm × 3.1 cm × 4.5 cm
EMG acquisition box weight	46 g
Principle	Differential voltage
Number of electrodes	3
Bandwidth	10–400 Hz
Gain	1000
Input impedance	100 GΩ
CMRR	110 dB
Operating voltage	*V_cc_* = 3.3 V

The EMG signal acquired through the developed one channel interface is used to control the stiffness of each module of the robotic device through the implemented control scheme based on servo motor.

In the following, we will explain how stiffness regulation has been obtained using servomotors. Generally, in active compliance control framework, the equation relating the motor torque to its position is given by

$$\tau =k\Delta_{q}=k(q_{des}-q_{m})$$

where *q_des_* is the desired (reference) joint position, *q_m_* is the measured (current) joint value, and *k* is the stiffness constant (Siciliano et al., 2010). Note that the compliant (or stiff) behavior of the joint is achieved by virtue of the control, differently from what happen in mechanical systems with a prevalent dynamics of elastic type. This controller is typically used with actuator that can be torque controlled. Servo motors are position controlled actuators where it is not possible to directly command the exerted torque. A small reference position variation in the clockwise direction is counterbalanced by a large amount of torque in the counterclockwise direction to compensate for this. This behavior is regulated by the controller embedded in the servo motor and cannot be modified. This torque–position relationship defines the standard stiffness of the servo motor (*k_c_*) that cannot be changed by the user. The only servomotor parameter that can be commanded is its desired position *q_des_*. We considered that, at time instant *t*, the desired position for the *i*-th servomotor is obtained as

$$q_{des,i}(t)=q_{m,i}(t-1)+\Delta q_{i}(t-1),$$

where

(1) $$\Delta q_{i}(t)=k_{d}k_{c}(q_{des}-\;q_{m}).$$

The scaling factor *k_d_* is introduced to modulate the position error. In order to vary the parameter *k_d_*, we used the EMG signal acquired at the user biceps. In particular, the range of EMG signals was linearly mapped in the range 0.4–3 of parameter *k_d_*. In Figure 5B, a plot of the relation between biceps contraction and commanded displacement is reported for one module. In presence of a rigid grasped object, the measured positions of the extra finger joints do not change due to the object constraints. So that, changing the desired position of the servomotors through the scaling factor, we can control the force exerted by the device onto the object. In other words, changing the value of *k_d_*, it is possible to command a position of the module that results in a higher force applied onto the object.

In order to regulate the stiffness between modules, we set priorities. We considered two distinct cases. If only the fingertip module is in contact with the object, all the other modules change their stiffness accordingly. This solution allows to control the stiffness of modules that are not in contact with the object in precision grasps. In power grasps, in order to obtain suitable contact points, we set different priorities according to the position of the module in the finger. If the fingertip module comes in contact first, the remaining modules change their stiffness accordingly. If another module comes in contact first, modules below to it regulate their stiffness, while the module above no. The same methodology is followed for other intermediate modules. The contact of a module is detected comparing the desired angle commanded to the servo motor (*q_des_*) with the actual position read by the encoders (*q_m_*). When Δ*q* = ||*q_des_* − *q_m_*|| overtake a predefined threshold a contact is recognized. After the contact is achieved, the compliance can be regulated.

#### 2.2.2 EMG Armband Gesture Recognition and Robotic Device Motion Control

We used a Myo Armband at forearm to recognize the hand gestures that control the device motions. This device has electrically safe setup with low voltage battery and Bluetooth LE protocol, eight surface EMG sensors working at frequency of 2200 Hz and 9-DoF IMU working at 50 Hz. The provided software development kit (SDK) is suitable for working with the recorded data and for developing standalone applications. EMG signals are filtered through notch filters at frequencies of 50 and 60 Hz in order to take out any power-line interference. For the sake of simplicity, we considered the five gestures available with the SDK. These gestures mainly involve flexion/extension of fingers and flexion/extension of hand.

**Figure 6:**
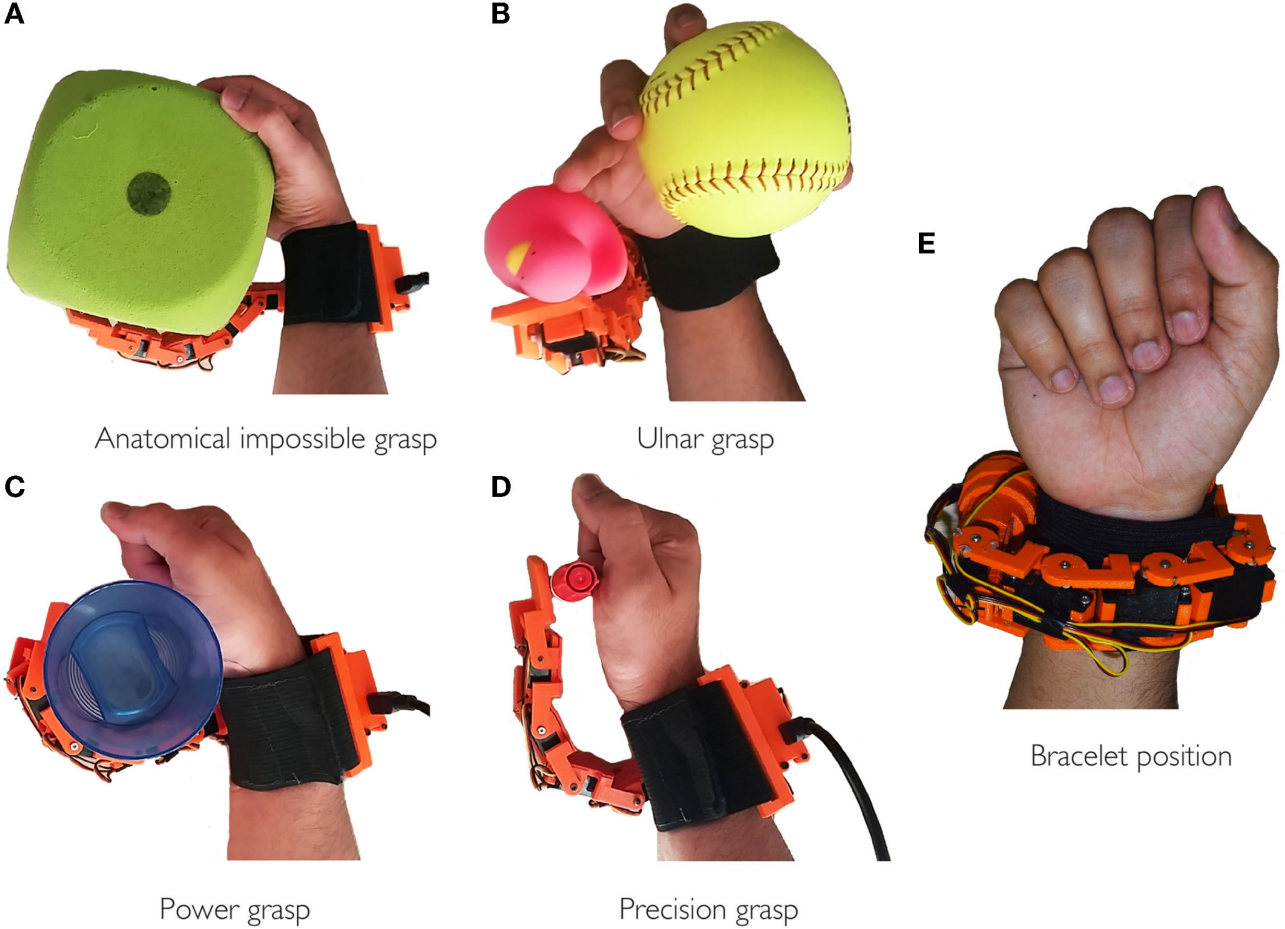
**Examples of possible achievable grasps at working positions (A–D) and bracelet at rest position (E)**. In **(A,B)**, the robotic finger coordinates with healthy hand to realize the *anatomically impossible* and *ulnar* grasp, respectively. While in **(C,D)**, it interacts with paretic hand to realize *power* and *precision grasp*.

**Figure 7:**
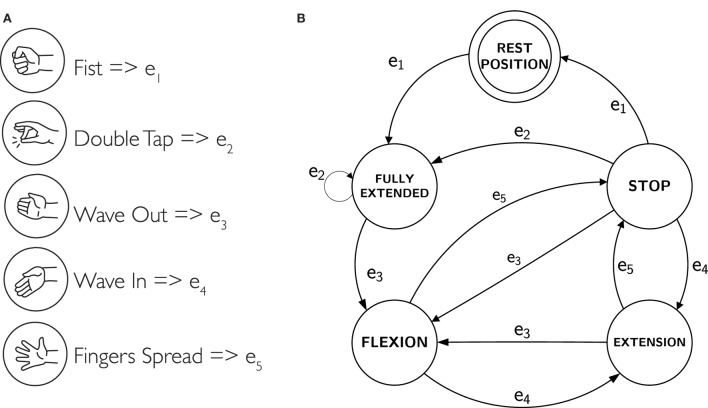
**(A)** The recognized gestures and associated trigger signal. **(B)** The finite state machine that controls the motion of the robotic device in corresponds to the generated gesture.

We implemented specific types of grasps for both kinds of users in order to make better suitable to use the robotic finger with healthy hand or paretic hand. In particular, in case of healthy hand, we defined *anatomically impossible* grasps and ulnar grasps (see Figures 6A,B). In case of *anatomically impossible* grasp, the supernumerary robotic finger coordinates with human hand to grasp big size objects that cannot be grasped using only one hand. In *ulnar* grasp configuration, the robotic device coordinates with ring and pinkie fingers to grasp and hold an object, while the upper part of the hand (thumb, index, and medium fingers) is left free to do another task allowing, for instance, to hold multiple object in one hand or to unscrew a bottle cap with a single hand. In case of paretic hand users, we defined *power* and *precision* grasp as shown in Figures 6C,D. In the former, each module flexes with a fixed step size in order to wrap the finger around the object. In the latter, the target is to hold small size objects between the paretic limb and the device fingertip pad. To this aim, the fingertip is kept parallel to the paretic limb during flexion motion. The contact is expected to occur between the object and the fingertip module. Finally, the supernumerary robotic finger can actively be wrapped around the wrist as a bracelet when not used (see Figure 6E). We implemented a trigger-based FSM to control the motion of the robotic device (see Figure 7B). All the gestures were associated with a unique trigger signal. In Figure 7A, the gestures recognized through the Myo Armband are shown. In particular, *fist*-(event *e*_1_) switches the device from bracelet position to working position and vice versa. *Double tap*-(event *e*_2_) changes the grasp modalities. Patients with paretic hand can switch between precision and power grasp. When augmentation purpose is concerned, the user can switch between ulnar and anatomically impossible grasp. *Wave out*-(event *e*_3_) corresponds to flexion, and *Wave in*-(event *e*_4_) is associated with extension. Finally, *Finger spread*-(event *e*_5_) can stop the motion of the robotic finger.

## 3 Experiments

In Section 2, we introduced a novel EMG interface to control motion and compliance of a supernumerary robotic finger. In the following, we demonstrate how this interface and the wearable device can be effectively used both to compensate paretic hand functions and to augment healthy human hand capabilities. We performed a proof-of-concept study involving four healthy subjects (three male and one female, aged 29–40 years). Written informed consent was obtained from the participants. The procedures were in accordance with the Declaration of Helsinki. The aim of this study was to verify the potential of the approach and to understand how rapidly the subjects can successfully interact with the wearable device by using the proposed EMG control interface. The experiments were divided into two categories. The first set of experiments was related to compensation of grasping function, whereas the second was related to augmentation of hand capabilities. In particular, the compensation experiments, shown in Section 3.1, have been carried out asking to the subjects to simulate a paretic hand. We focused mainly on bimanual tasks of activity of daily living (ADL). The augmentation experiments shown in Section 3.2 were performed with the healthy hand to show the effectiveness of the device in increasing the hand grasping abilities and workspace, e.g., allowing to grasp big size objects which can not be grasped using a single hand or holding multiple objects using the augmented hand, i.e., human hand and the supernumerary robotic finger. In both the experimental sets, the subjects used the EMG interface on one arm (three subject used the right arm, one the left), whereas the supernumerary robotic finger was worn on the other arm. The Myo Armband was positioned at the forearm, while the one channel electrodes interface on the biceps (see Figure 2).

### 3.1 Compensation of Paretic Hand Functions

**Figure 8:**
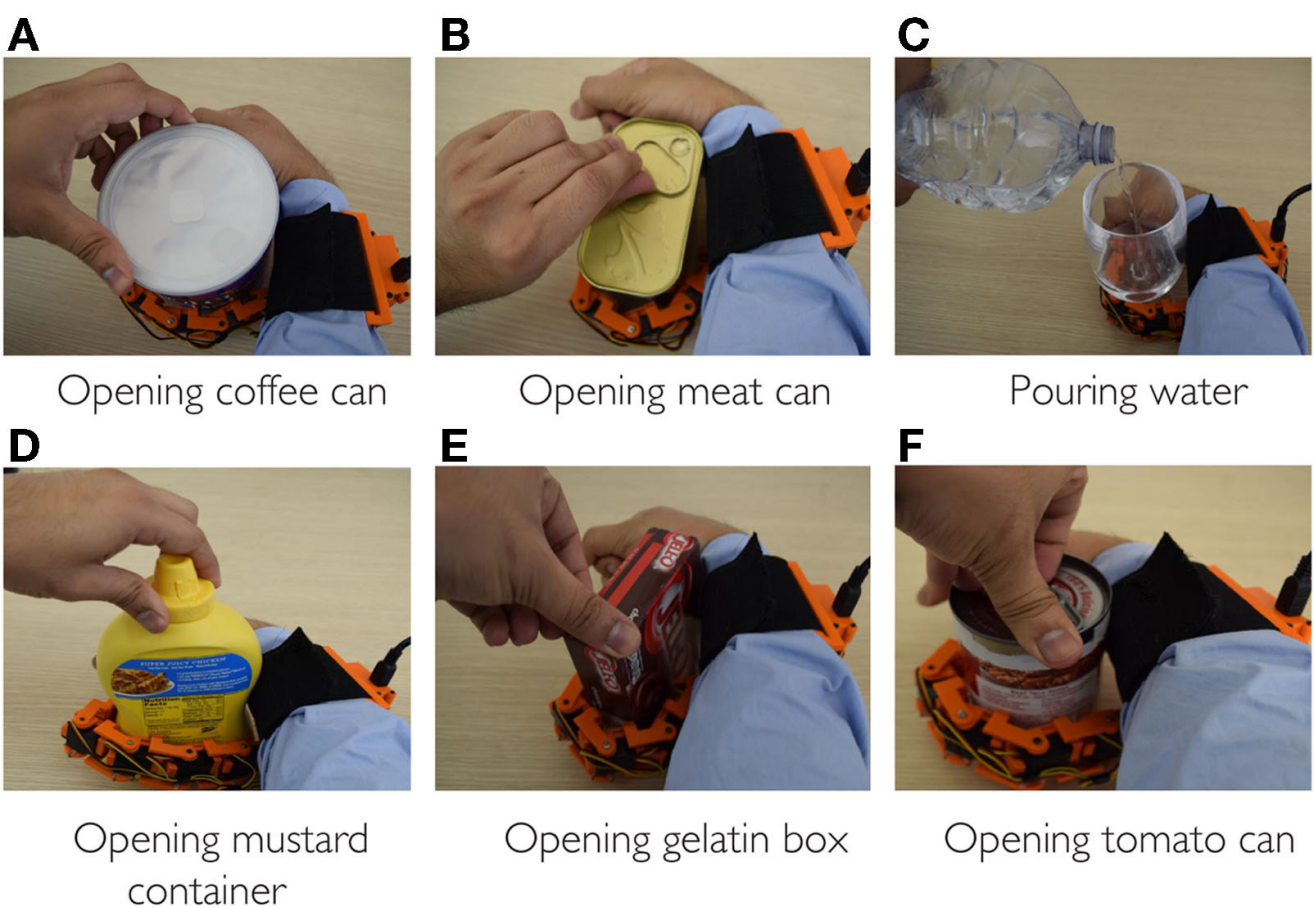
**Supernumerary robotic finger helping in bimanual task of ADL**. All the bimanual tasks can be completed in the presence of robotic device even if one hand is non-functional. **(A)** Opening coffee can, **(B)** opening meat can, **(C)** pouring water, **(D)** opening mustard container, **(E)** opening gelatin box, and **(F)** opening tomato can.

Among the different ADL, we focused on those involving “hold and manipulate” tasks. Such activities are generally bimanual tasks where one hand is used to restrain the motion of one object, while the other operates on it, e.g., unscrew the cap of a bottle, open a beans can, etc. The proposed supernumerary finger can be an effective aid in such tasks (Hussain et al., 2016). To demonstrate how the EMG interface can be used by patients, we asked to the subjects to execute different ADL involving a hold and manipulate task (see Figure 8). In particular, the subjects were asked to grasp an object using the gestures of the hand and to regulate the grasp tightness acting on the stiffness of the device. We used a subset of objects from the YCB grasping toolkit (Çalli et al., 2015). This toolkit is intended to be used to facilitate benchmarking in prosthetic design, rehabilitation research, and robotic manipulation. The objects in the set are designed to cover a wide range of aspects of the manipulation problem. It includes objects of daily life with different shapes, sizes, textures, weight, and rigidity. We considered six objects with different shapes to show how the robotic finger can adapt to the shape of the objects to realize a stable grasp. We mainly targeted the objects used in kitchen and in other ADL. During all the tests, subjects simulated the paretic hand and device was positioned on the arm as supposed to be used with the patients.

The subject was asked to perform different bimanual ADL without using the hand grasping ability where the device was worn. The controlateral arm was always used to control the device motion and joints stiffness. Figure 8 shows the ADL tasks performed by simulating a paretic hand. All the targeted tasks normally require two healthy hands but have been successfully executed with the aid of the robotic extra finger even if one hand was non-functional. The robotic finger and paretic hand was used to constrain the object, while healthy hand was used for manipulation. Figures 8A,B,D–F show the example of opening the cans, box, and bottle with various shapes and different caps. Figure 8C reports the task of pouring water from a bottle while holding the glass with the help of robotic device and paretic arm. All the task were fulfilled controlling the device through the proposes’ interface. The subjects used hand gestures to shape the finger around the object. Later, they controlled the grasp stiffness by contracting the controlateral arm biceps. Note that all the “opening” tasks required stiffness control to be executed. In fact, while compliant joints are preferable to adapt the shape of the finger to the object to grasp, a stiff device is necessary to achieve the stable grasps necessary while unscrewing the caps.

### 3.2 Augmenting Healthy Hand Function through the Proposed System

**Figure 9:**
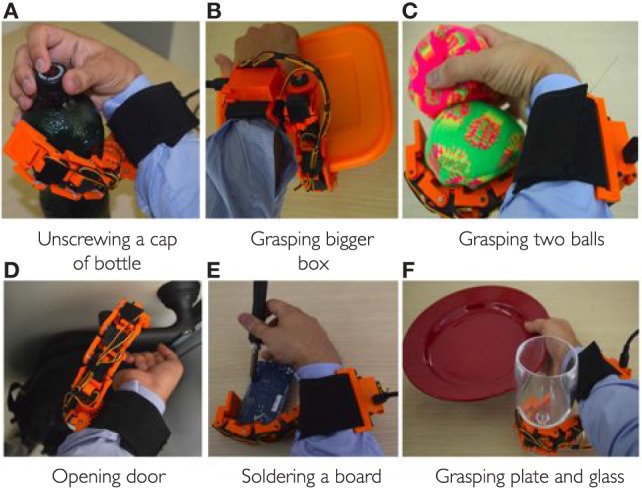
**Examples of tasks performed by the augmented hand, i.e., human hand plus supernumerary robotic finger**. In all the tasks, the human healthy hand and robotic finger work together to complete the tasks that are impossible to do with human hand only. **(A)** Unscrewing a cap of bottle, **(B)** grasping bigger box, **(C)** grasping two balls, **(D)** opening door, **(E)** soldering a board, and **(F)** grasping plate and glass.

In this experiment, the subjects were asked to grasp a set of objects with the augmented hand to prove the effectiveness of the extra-robotic finger in enlarging the human hand workspace and dexterity. We targeted tasks involving either *anatomically impossible* grasp or *ulnar* grasp, as defined in Section 2. In the former case, the subjects were asked to grasp relatively big size objects which cannot be grasped using only one hand. In the latter case, the users tried to grasp objects only using the ring and the pinkie fingers opposite to the sixth finger and to perform another operation with the remaining fingers (thumb, index, and middle). In Figure 9A, the user is unscrewing a cap from a bottle using only one hand. Ulnar grasp is used to keep firm the bottle, while the other fingers can unscrew the cap. Figure 9B shows the example of grasping big size box with the augmented hand that is impossible to grasp with the human hand only. Holding multiple objects with the augmented hand is shown in Figures 9C,F. The example illustrated in Figure 9D involves the task of opening the door using the handle, while carrying a heavy bag with the hand. The user was able to turn the handle to open the door using the robotic device, while keep holding the bag with the hand. The 9-e is another example where the user can solder a circuit board, while holding the board by robotic finger, ring, and pinkie. The thumb, index, and middle finger are used to hold soldering gun. Note that, all the tasks are either impossible or at least very difficult to be carrying out with a single hand. All these tasks were successfully fulfilled by all the subjects with the help of the EMG interface and the supernumerary extra finger. Also in this subset of examples, the possibility to control both motion and joint stiffness of the device was exploited by the users.

## 4 Results

**Figure 10:**
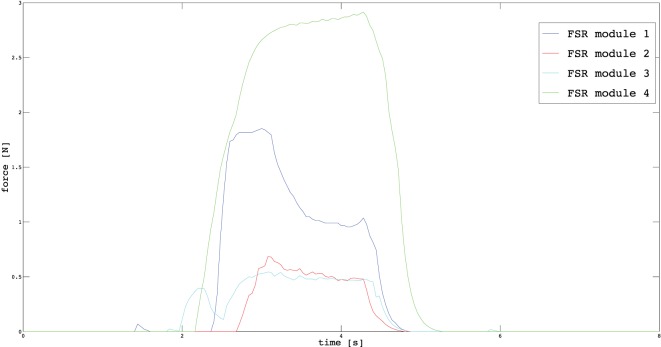
**Forces exerted by the modules on the grasped object during a power grasp**.

**Figure 11:**
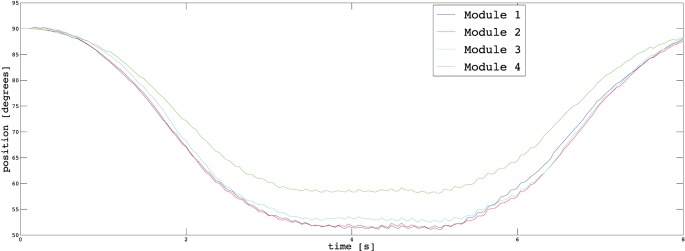
**Positions of the modules during a power grasp**.

**Figure 12:**
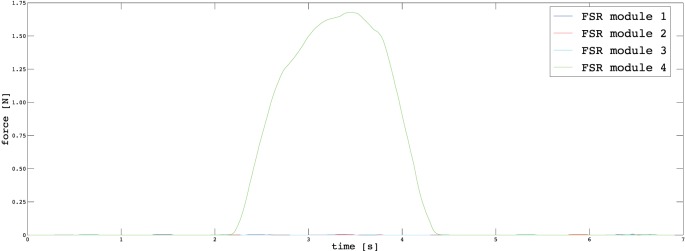
**Forces exerted by the modules on the grasped object during a precision grasp**.

**Figure 13:**
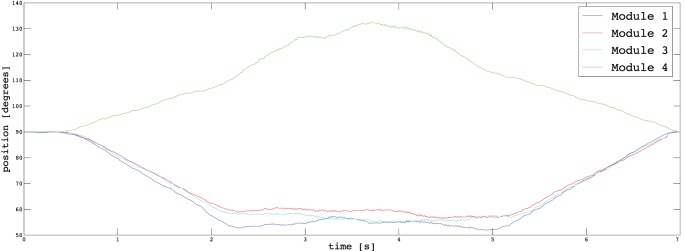
**Positions of the modules during a precision grasp**.

**Figure 14:**
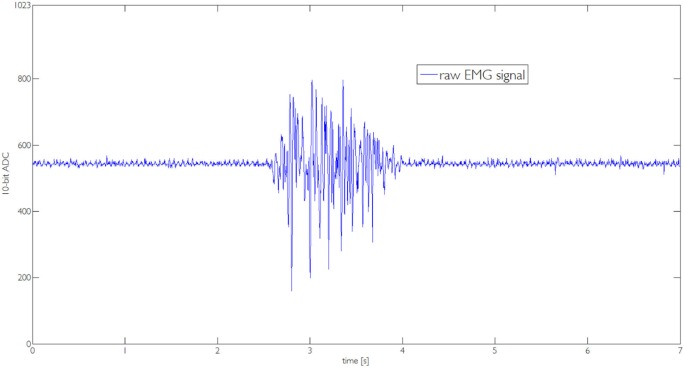
**Raw EMG signal captured by the one channel interface during the execution of the task reported in Figure 8A**.

**Figure 15:**
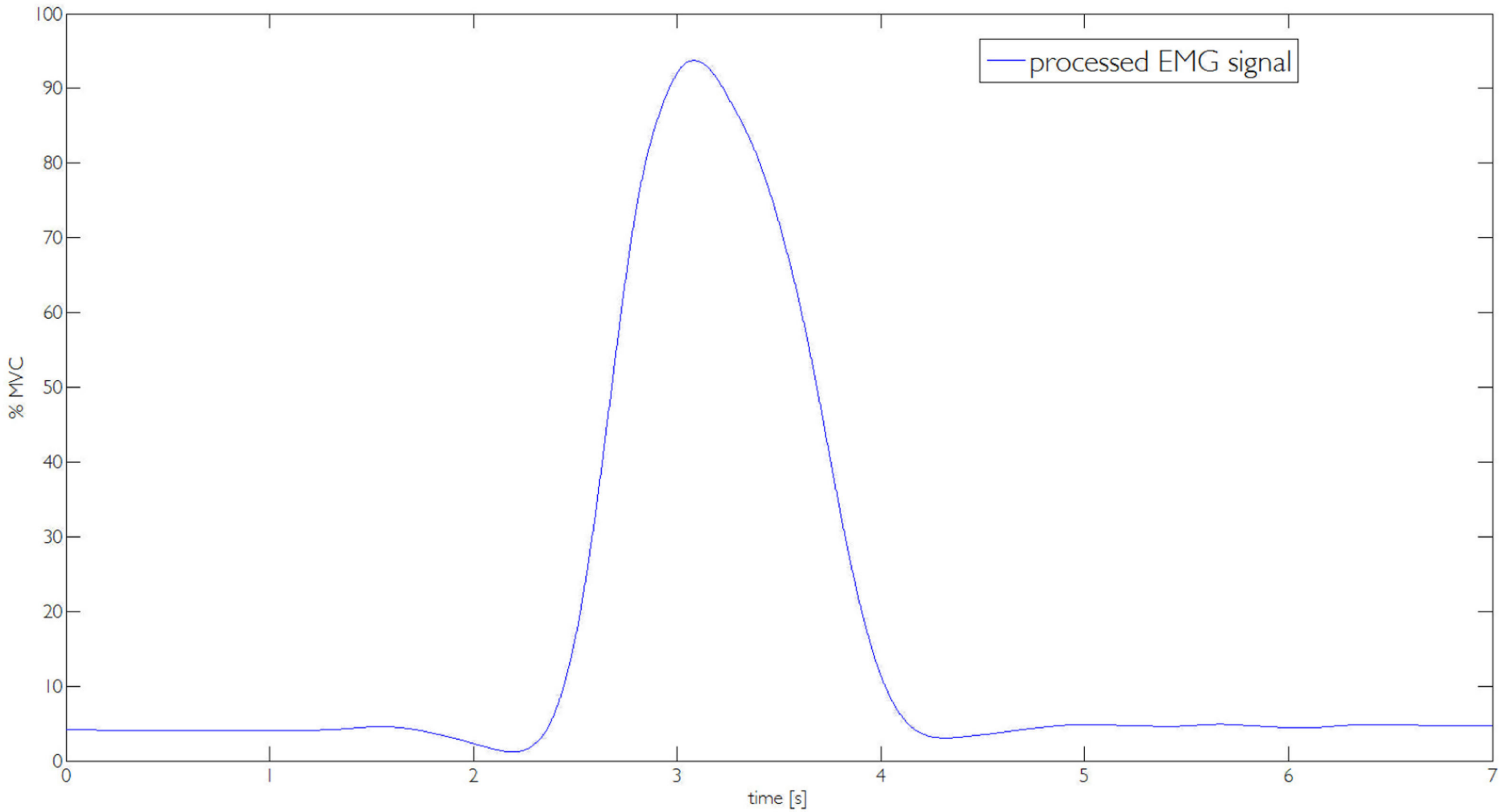
**The processed EMG signal used to compute the value of parameter *k_d_***.

In Section 3, we described the tasks performed by the subjects to prove the usability of the proposed EMG interface and the novel supernumerary finger prototype. In the following, we will give the details of the position of the device and the forces exerted on the grasped object for two particular type of grasps, i.e., power and precision grasps. Figures 10–13 reported the behavior of the device during power and precision grasping, respectively. In particular, Figures 10 and 11 refer to the power grasp reported in Figure 8A, whereas Figures 12 and 13 refer to the precision grasp reported in Figure 9E. We report only these examples for the sake of brevity. Figures 10–13 represent the average of five repetitions of the same subject. To measure the forces exerted on the objects, we equipped each module of the extra finger with a Force Sensing Resistor (FSR) (408, Interlink Electronics Inc., USA). The user was asked to command the supernumerary finger till the grasp is obtained. Once the device was in contact with the object, the user increased the stiffness of the device by cocontracting his/her biceps (see Figure 14). The contraction of the biceps was read by the EMG interface, and the value of *k_d_* in equation (1) was increased (see Figure 15). This variation produced a variation in the desired angle *q_des_* of the modules, while the read actual position of the modules remained the same due to the constrain imposed by the object (see Figures 11 and 13). The variation in the desired angles produces, however, an increase of the force exerted by the device onto the object, as shown in Figures 10 and 12. So that, by cocontracting the biceps the user can regulate the grasp tightness. As expected, in power grasps, all the modules move of a similar angle so as to wrap the object. All the modules also contribute to the grasp tightness applying force on the object. Differently, in precision grasp, the fingertip module is the only module exerting force. The module motion is opposed to the direction of the other three modules so as to leave the fingertip parallel to the hand.

After the experiments, we investigated the users’ subjective satisfaction and possible concerns related to the proposed system. We proposed a questionnaire to the subjects to evaluate their satisfaction and usefulness of the proposed system. Questionnaires and interviews are recommended methods for user feedback and what features they particularly like or dislike in the system (Nielsen, 1994). The subjects were asked to fill the Usefulness Satisfaction and Ease of use questionnaire (USE) (Lund, 2001) that focuses on the experience of the system usage. This questionnaire uses a seven-point Likert rating scale. Mean and SD of the questionnaire factors are presented in Table 3.

**Table 3:** **Questionnaire factors and relative marks**.

Questionnaire factors	Mean (SD)
Usefulness	4.9 (0.6)
Ease of use	6.0 (0.5)
Ease of learning	6.3 (0.5)
Satisfaction	5.3 (0.5)

*The mark ranges from “1 = strongly disagree” to “7 = strongly agree.” Mean and SD are reported*.

The proposed EMG interface and the novel robotic extra finger prototype successfully enabled the users to complete all the targeted tasks both related to augmentation and compensation. The experiments proved that the presented system can be an effective aid both in augmenting the healthy human hand and in compensating its missing abilities in case of a disease. The proposed EMG control interface resulted to be intuitive and simple. The users were able to generate multiple control inputs without using sensorized gloves on human hand and were able to modulate the compliance of the robotic device in proportional to the EMG signal amplitude variations in biceps. Moreover, the upgraded version of the device with additional adduction/abduction degree of freedom increased the dexterity of the robotic device allowing more complex operation, especially when hand augmentation was considered.

## 5 Discussion

Supernumerary robotic limbs are a new generation of wearable robots which aims at assisting natural limbs by closely interacting with them. In order to realize safe and natural interaction of human limbs with the extra-robotic limbs, the control principles, actuation, and sensing capabilities of extra limbs should have similar behavior as humans ones, e.g., their ability to regulate compliance. In this regard and to overcome the limitations of the control interfaces presented in state of the art for supernumerary robotic fingers, we propose a novel EMG interface. In particular, to obtain multiple user control inputs to control the motion of extra-robotic finger, as well as to regulate its compliance, we have presented an EMG-based control interface that can be used to control different trajectories for finger flexion/extension and can regulate the finger compliance and thus the tightness of the grasp. The exploitation of the supernumerary robotic fingers in compensating and augmenting the human hand grasping abilities is at an early stage. One of the major challenges in augmenting/compensating human capabilities through robotic extra limbs concerns the development of a suitable control interfaces for the integration of the device motion with that of the human. We better demonstrate this fact by recalling the approaches presented in literature and their limitations. Wu and Asada (2014) presented a control algorithm enabling a human hand augmented with two robotic fingers to share the task load together and adapt to diverse task conditions. Postural synergies were found for the seven-fingered hand comprised of two robotic fingers and five human fingers through the analysis of measured data from grasping experiments. In Prattichizzo et al. (2014b), a mapping algorithm able to transfer to an arbitrary number of robotic extra fingers the motion of the human hand has been presented. The mapping algorithm was based on the definition of a virtual object obtained as a function of a set of reference points placed on the augmented hand (human hand and robotic fingers). The mapping algorithm allowed to move the extra fingers according to the human hand motions without requiring explicit command by the user. Both the approaches used an instrumented glove to track the human hand presenting some limitations which affected their practical application. Patients with a paretic hand cannot properly control finger motions, thus a dataglove interface cannot be used. The estimation of the human hand posture and fingers motion implies a reliable and computationally expensive hand tracking. Moreover, datagloves can be only used for position control of the robotic device without having any control on force or stiffness regulation. As a preliminary solution to the above mentioned issues, we implemented a trigger-based control approach (Hussain et al., 2015a,b). The trigger signal was activated by a wearable switch placed on a ring. A single switch activation regulated the stop/motion of the finger along a predefined flexion trajectory, while a double activation switched from flexion to extension and vice versa. Although the ring-based control approach resulted simple and intuitive, this control interface involved human hand thumb, thus, limiting the use of thumb in completion of tasks. Moreover, it offers few user control inputs to control the motion of the robotic finger and force control is not straightforward. The control approach and the device presented in this paper are a possible solution of the above mentioned issues of the techniques presented in literature. In Section 3, we reported several tasks where a supernumerary finger can be used both for grasping compensation of paretic limb and to augment human hand capabilities. In Section 4, we showed how the EMG interface can be effectively used to control the position of the finger and the force exerted on the object.

All the experiments were performed involving healthy subjects. We are currently starting to test the system with stroke patients showing a residual mobility of the arm. We delineate the patients’ condition for being included in the pilot experiments. Patients have to score ≤2 when their motor function is tested with the National Institute of Health Stroke Scale (NIHSS) (Brott et al., 1989), item 5 “paretic arm.” Moreover, the patients has to show the following characteristics: (1) normal consciousness (NIHSS, item 1a, 1b, 1c = 0), absence of conjugate eyes deviation (NIHSS, item 2 = 0), absence of complete hemianopia (NIHSS, item 3 ≤ 1), absence of ataxia (NIHSS, item 7 = 0), absence of completely sensory loss (NIHSS, item 8 ≤ 1), absence of aphasia (NIHSS, item 9 = 0), absence of profound extinction and inattention (NIHSS, item 11 ≤ 1).

## 6 Conclusion

In this paper, we present an EMG control interface for a supernumerary robotic finger that can be used to control motion and joint stiffness. The aims are grasping compensation in chronic stroke patients and augmentation of human healthy hand to enhance its grasping capabilities and workspace. The motion of the robotic finger is controlled through gesture recognition and its compliance is regulated by EMG signal amplitude variations. In particular, we proposed Myo Armband to recognize the user gesture to control the motion of the robotic device. We developed EMG one channel electrode interface to modulate the compliance of the robotic device through a control scheme based on servo motor. We developed a five DoFs device that can be worn on the user wrist by an elastic band. We validated the use of device in augmenting and compensating the human hand grasping abilities. In particular, we showed how the supernumerary robotic finger can play the role of an extra thumb enlarging the human hand workspace and the hand dexterity and how it can compensate the missing abilities of the non-functional hand in case of stroke patients. We demonstrate through experiments that the same interface can be used by patient and healthy subjects to control different flexion trajectories and to regulate the grasp tightness.

As future work, we are improving the portability of the system, in particular, we are realizing a Bluetooth communication of EMG interfaces with the robotic device controller. We are also testing the EMG interface with stroke patients so as to collect interesting insights for the extra finger development.

## Author Contributions

IH contributed in literature review, design, and development of robotic finger; implementation of control, calibration, testing, experiments, and paper writing. GSp contributed the development of EMG interfaces design and development. GSa contributed in control development and in paper writing. DP supervised the project overall and contributed in writing and proof reading.

## Conflict of Interest Statement

The authors declare that the research was conducted in the absence of any commercial or financial relationships that could be construed as a potential conflict of interest.

## References

[B1] Ajoudani A. Tsagarakis N. G. Bicchi A. (2012). “Tele-impedance: towards transferring human impedance regulation skills to robots,” in IEEE International Conference on Robotics and Automation (ICRA), 2012 (Saint Paul, MN: IEEE), 382–388.

[B2] Brott T. Adams H. Olinger C. P. Marler J. R. Barsan W. G. Biller J. (1989). Measurements of acute cerebral infarction: a clinical examination scale. Stroke 20, 864–870. 10.1161/01.STR.20.7.871 2749846

[B3] Çalli B. Walsman A. Singh A. Srinivasa S. Abbeel P. Dollar A. M. (2015). Benchmarking in manipulation research: the YCB object and model set and benchmarking protocols. CoRR abs/1502.03143. Available at: http://arxiv.org/abs/1502.03143

[B4] Carrozza M. C. Suppo C. Sebastiani F. Massa B. Vecchi F. Lazzarini R. (2004). The spring hand: development of a self-adaptive prosthesis for restoring natural grasping. Auton. Robots 16, 125–141. 10.1023/B:AURO.0000016863.48502.98

[B5] Davenport C. Parietti F. Asada H. H. (2012). “Design and biomechanical analysis of supernumerary robotic limbs,” in ASME 2012 5th Annual Dynamic Systems and Control Conference Joint with the JSME 2012 11th Motion and Vibration Conference (American Society of Mechanical Engineers), 787–793.

[B6] Farina D. Merletti R. (2000). Comparison of algorithms for estimation of emg variables during voluntary isometric contractions. J. Electromyogr. Kinesiol. 10, 337–349. 10.1016/S1050-6411(00)00025-0 11018443

[B7] Heo P. Gu G. M. Lee S.-J. Rhee K. Kim J. (2012). Current hand exoskeleton technologies for rehabilitation and assistive engineering. Int. J. Precis. Eng. Manuf. 13, 807–824. 10.1007/s12541-012-0107-2

[B8] Hogan N. (1985). Impedance control: an approach to manipulation: part I, II, and III. J. Dyn. Syst. Meas. Control 107, 8–16. 10.1115/1.3140713

[B9] Hussain I. Meli L. Pacchierotti C. Salvietti G. Prattichizzo D. (2015a). “Vibrotactile haptic fedback for intuitive control of robotic extra fingers,” in Proc. IEEE World Haptics Conference (WHC) (Chicago, IL).

[B10] Hussain I. Salvietti G. Meli L. Pacchierotti C. Cioncoloni D. Rossi S. (2015b). “Using the robotic sixth finger and vibrotactile feedback for grasp compensation in chronic stroke patients,” in IEEE International Conference on Rehabilitation Robotics (ICORR) (Singapore: IEEE), 67–72.

[B11] Hussain I. Salvietti G. Spagnoletti G. Prattichizzo D. (2016). The soft-sixthfinger: a wearable emg controlled robotic extra-finger for grasp compensation in chronic stroke patients. IEEE Robot. Autom. Lett. 1, 1000–1006. 10.1109/LRA.2016.2530793

[B12] Jones L. A. Lederman S. J. (2006). Human Hand Function. Oxford: Oxford University Press.

[B13] Llorens-Bonilla B. Parietti F. Asada H. H. (2012). “Demonstration-based control of supernumerary robotic limbs,” in IEEE/RSJ International Conference on Intelligent Robots and Systems (IROS) (Vilamoura: IEEE), 3936–3942.

[B14] Lum P. S. Godfrey S. B. Brokaw E. B. Holley R. J. Nichols D. (2012). Robotic approaches for rehabilitation of hand function after stroke. Am. J. Phys. Med. Rehabil. 91, S242–S254. 10.1097/PHM.0b013e31826bcedb 23080040

[B15] Lund A. M. (2001). Measuring usability with the use questionnaire. Usabil. Interface 8, 3–6.

[B16] Merletti R. Botter A. Troiano A. Merlo E. Minetto M. A. (2009). Technology and instrumentation for detection and conditioning of the surface electromyographic signal: state of the art. Clin. Biomech. 24, 122–134. 10.1016/j.clinbiomech.2008.08.006 19042063

[B17] Merlo A. Campanini I. (2010). Technical aspects of surface electromyography for clinicians. Open Rehabil. J. 3, 98–109. 10.2174/1874943701003010098

[B18] Nakayama H. Jorgensen H. S. Raaschou H. O. Olsen T. S. (1994). Compensation in recovery of upper extremity function after stroke: the copenhagen stroke study. Arch. Phys. Med. Rehabil. 75, 852–857. 10.1016/0003-9993(94)90161-9 8053790

[B19] Nielsen J. (1994). Usability Engineering. Elsevier.

[B20] Pons J. L. (2008). Wearable Robots: Biomechatronic Exoskeletons, Vol. 338 Wiley Online Library Available from: http://www.wiley.com/WileyCDA/WileyTitle/productCd-0470512946.html

[B21] Prattichizzo D. Malvezzi M. Hussain I. Salvietti G. (2014a). “The sixth-finger: a modular extra-finger to enhance human hand capabilities,” in Proc. IEEE Int. Symp. in Robot and Human Interactive Communication (Edinburgh, UK).

[B22] Prattichizzo D. Salvietti G. Chinello F. Malvezzi M. (2014b). “An object-based mapping algorithm to control wearable robotic extra-fingers,” in Proc. IEEE/ASME Int. Conf. on Advanced Intelligent Mechatronics (Besançon, France).

[B23] Salvietti G. Hussain I. Cioncoloni D. Taddei S. Rossi S. Prattichizzo D. (2016). Compensating hand function in chronic stroke patients through the robotic sixth finger. IEEE Trans. Neural Syst. Rehabil. Eng. PP, 1–1. 10.1109/TNSRE.2016.2529684 26890911

[B24] Siciliano B. Sciavicco L. Villani L. Oriolo G. (2010). Robotics: Modelling, Planning and Control. Springer Science & Business Media.

[B25] Taylor C. L. Schwarz R. J. (1955). The anatomy and mechanics of the human hand. Artif. Limbs 2, 22–35. 13249858

[B26] Wu F. Asada H. (2014). “Bio-artificial synergies for grasp posture control of supernumerary robotic fingers,” in Proceedings of Robotics: Science and Systems (Berkeley, CA).

